# Evaluation of membrane bioreactor for advanced treatment of industrial wastewater and reverse osmosis pretreatment

**DOI:** 10.1186/2052-336X-11-34

**Published:** 2013-12-19

**Authors:** Majid Hosseinzadeh, Gholamreza Nabi Bidhendi, Ali Torabian, Naser Mehrdadi

**Affiliations:** 1 Graduate Faculty of Environment, University of Tehran, Tehran, Iran

**Keywords:** Membrane bioreactor, Reverse osmosis, Silt density index, Fouling

## Abstract

The evaluation of a membrane bioreactor (MBR) for pretreatment of reverse osmosis (RO) in order to reuse and reclamation of industrial town wastewater treatment plant was investigated in this study. Performance of MBR effluent through water quality in term of parameters such as chemical oxygen demand (COD), total suspended solids (TSS), total nitrogen (TN) and total coliform (TC) were measured. Also Silt density index (SDI) was used as indicator for RO feed water. The results of this study demonstrated that MBR produce a high quality permeate water. Approximately 75%, 98%, 74% and 99.9% removal of COD, TSS, TN and TC were recorded, respectively. Also SDI of the permeate effluent from membrane was below 3 for most of the times. It means that pilot yield a high quality treated effluent from the membrane module which can be used as RO feed water.

## Introduction

Nowadays water resources are becoming increasingly scarce in many areas of the world particularly in arid regions such as Middle East due to factors such as increasing population, climate changes, industrial development and increasing water use per capita, etc. Because of the scarcity of water resources, wastewater reclamation and reuse is an effective tool for sustainable industrial development program in both developing and developed countries. Currently, treated industrial wastewater is typically discharged to the environment in most industrial towns in Iran. These effluents have a potential to reclamation and reuse for produce industrial process water. For reach this reuse application further treatment would be needed. Nowadays membrane separation processes are becoming quite popular in wastewater treatment and reclamation, since they combine process stability with an excellent effluent quality [1-4]. One of this membrane processes for water reuse and reclamation is reverse osmosis (RO) that is increasingly being used in all over the world [5-9]. The main problem of using RO is its membrane fouling that is prevalent in water reclamation applications. In fact membrane fouling is the main cause of permeate flux decline and loss of product quality in reverse osmosis Systems [10,11]. Sources of fouling can be divided into four principal categories: scaling, particular fouling, bio fouling and organic fouling. To avoid common issues that can result in system failure, RO systems need to be coupled with an effective pretreatment.

MBR has been widely studied and applied on full scale in wastewater treatment and it is considered as a new pretreatment for reverse osmosis in water reclamation and reuse among many different pretreatment schemes for RO [12-18].

MBR is a process in which conventional biological system is coupled with the membrane process. There are two configurations for MBRs which are in-series and submerged MBRs which submerged membrane bioreactors have lower power requirements than the in-series configurations [19,20].

Due to the shortage of water resources in the Shokouhieh industrial town (located in Qom province, Iran) reclamation and reuse of industrial wastewater treatment plant effluent using RO modules was put on the agenda. Effluents of this WWTP were not being adequately treated by biological treatment and there are biodegradable organic matters in effluent of wastewater treatment plant. This research has focused on the evaluation of the pilot scale operation and monitor of an MBR system to advance treatment of an industrial wastewater treatment plant effluent in order to produce water with appreciate quality as RO feed water. In other words this study has discussed the feasibility of RO pretreatment for water reuse from industrial wastewater treatment effluent (before disinfection) with operation of a MBR pilot. The removal of certain pollution parameters such as chemical oxygen demand (COD), suspended solids (SS), total nitrogen (TN) and total coliform (TC) were monitored.

## Material and methods

### Effluent source

Actual effluent used in this study as was taken from outlet of an industrial wastewater treatment plant of Shokouhieh, Qom, Iran. This plant receives and treats the wastewater from different factories such as welding, dairy, beverage, metal finishing, etc. Main units of Shokouhieh treatment plant are screens, an equalization tank, an anaerobic reactor, an aeration aerobic tank, sedimentation, sand filter and a disinfection system. Due to poor design, this existing treatment system is not effective in removing the all organic load of influent wastewater. Therefor there is significant amount of biodegradable organic matters in effluent. The effluent samples as MBR feed wastewater were collected from outlet of sand filters in plastic containers and were delivered to the laboratory where pilot is operated there. The typical physicochemical characteristics of the effluent are presented in Table 1. Values for parameters in Table 1 were measured from wastewater treatment plant effluent stream line after sand filters and before chlorination unit.

**Table 1:** Shokouhieh effluent characteristics

**Parameter**	**Unit**	**Value**
pH		7.3 ± 0.62
SS	mg/L	223 ± 32
COD	mg/L	250 ± 64
T-N	mg/L	51 ± 30
TC	MPN/100 mL	1.75×10^6^ ± 35×10^4^

### MBR pilot unit

**Figure 1:**
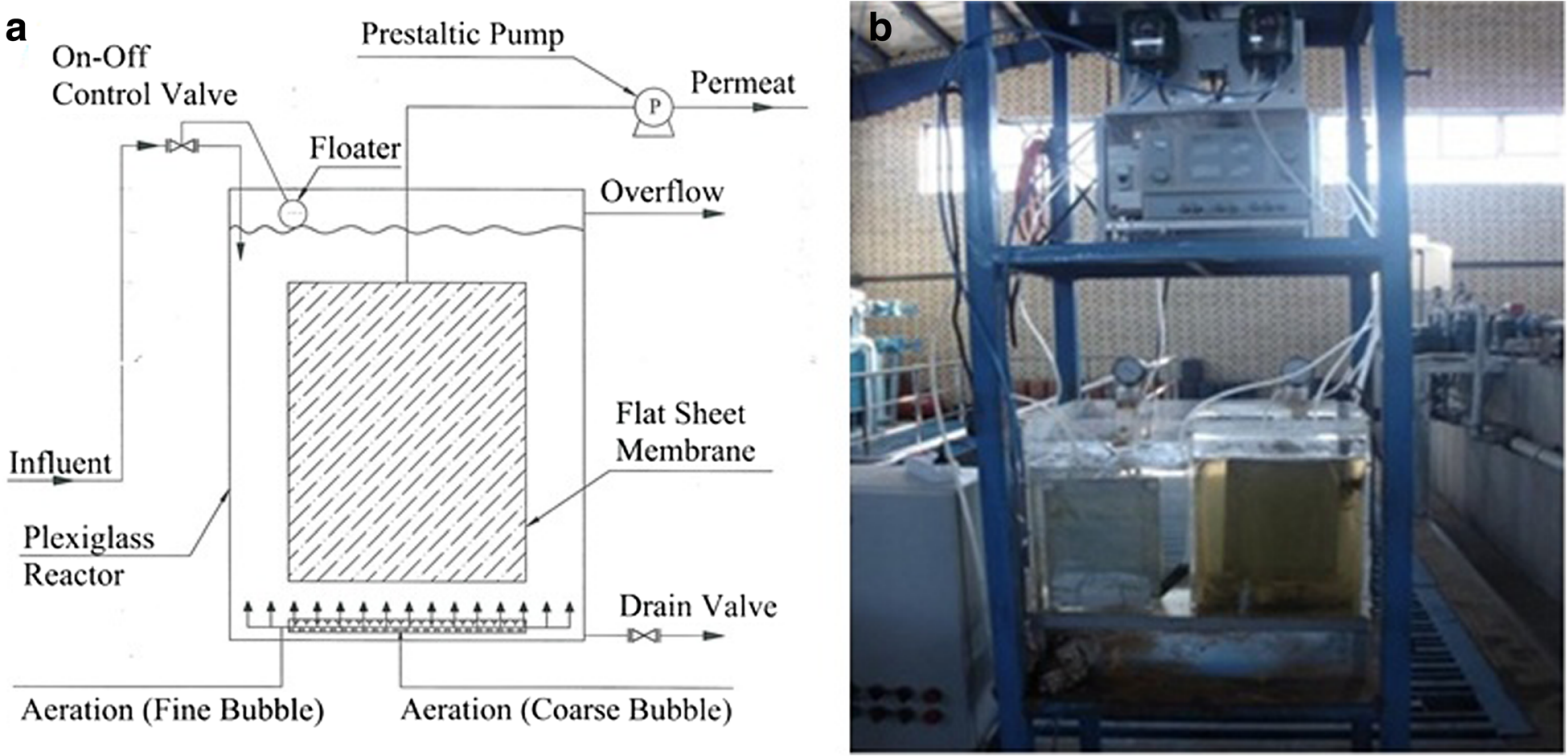
Membrane bioreactor pilot scale in Shokouhieh wastewater treatment plant: (a) Schematic process flow diagram; (b) picture of MBR module in operation.

Continuous operation of a pilot scale ultrafiltration membrane bioreactor system was carried out in this study. Schematic process flow diagram of pilot set up with a picture of system in operation is shown in Figure 1.

The bioreactor was made of Plexiglass with total volume of 32 liters. A flat sheet membrane ultrafilter was placed in the center of bioreactor. The membrane specifications are summarized in Table 2.

**Table 2:** Specification of membrane

**Process parameters**	**Unit**	**Value**
Membrane configuration		Flat sheet
Cut off	kDalton	150
Pore size	μm	0.4
Dimensions (Width × Height)	mm	240×200
Effective surface area	m^2^	0.048
Material	-	EPS
Membrane charge	-	Neutral
pH resistance range	-	4-11

Membrane was operated at a constant flow rate of 4 L/hr using a prestaltic pump. Air blower was used to provide required sufficient air during operating the MBR. Air was introduced via perforated plastic tube air diffusers which were located at the bottom of the reactor to produce fine and coarse bubbles for supply dissolved oxygen required for biological process in the reactor and reduce fouling on the membrane, respectively. Also pilot was equipped with control instruments for measuring temperature, dissolved oxygen (DO), pH and wastewater level.

### Operating conditions

MBR operating characteristics are summarized in Table 3. Membrane bioreactor was operated continuously, corresponding to an 8-hour hydraulic retention time (HRT) and the duration of operation was 30 days. Prior to use, membrane was washed with tap water until a steady pure water permeate flux was obtained. During operation, MLSS temperature in the bioreactor was kept constant at 22–27°C with a heat exchanger. Permeate flux was set to approximately 83 L/m^2^/hr using a prestaltic pump and transmembrane pressure (TMP) was continuously recorded using an analogue pressure gage. Chemical cleaning of the membrane module was not carried out during the operation. No biomass was initially removed from the reactor to allow the biomass concentration build up in the system to about 2000 mg/L. After that daily withdrawal of mixed liquor was conducted from the reactor in order to maintain the predetermined SRT (25 day) and control increase of organic matter and solid concentrations in the bioreactor.

**Table 3:** Operation condition for MBR

**Process parameters**	**Unit**	**Value**
Mode of operation		continuous
Operating temperature	°C	22-27
Permeate flow rate	L/hr	4
Filtration flux	L/m^2^/hr	83
pH in reactor	-	6.4-7.8
HRT	hr	8
SRT	day	25
MLSS	mg/L	1700-2450
MLVSS	mg/L	1510-2230
Mixed liquor DO	mg/L	2-4

The membrane was cleaned chemically once the pressure reached about 60 kPa. In this stage after taking out the membrane, it was soaked in a 250 mg/L NaOCl solution and afterwards with 4000 mg/L citric acid solution for at least 4 hours. Then membrane was cleaned with tap water. Fine bubble aeration was provided to maintain the dissolved oxygen (DO) concentration in the biological reactor higher than 2 mg/L. Fine bubble aeration was accomplished using suitable diffusers placed at the bottom of the biological reactor. Also coarse bubble aeration was supplied to the membrane module to minimize membrane fouling.

### Analytical method

Laboratory analyses were conducted to determine the characteristics of influent wastewater to pilot, activated sludge and MBR permeate. For this, suspended solids, chemical oxygen demand, total nitrogen and total coliform were analyzed. Most analytical techniques used in this research followed the standard methods described by APHA [21]. Data in this paper was averaged by at least 2 experiment results at each process. MLSS was measured with a Whatman glass microfiber filter using APHA 2540E standard method. The COD content of the samples was measured using Hach COD reactor and TN of the samples was measured using the Merck TN kit. The determination of total coliform was carried out for MBR feed and permeate samples using standard method 9222 B procedure.

The temperature of inlet and outlet stream of MBR and MLSS temperature in the reactor was monitored using a digital temperature probe (JENWAY, England ). These data were verified periodically using an alcohol thermometer. Also MBR influent and effluent pH value were determined using a portable pH meter (JENWAY-370, England) and dissolved oxygen meter was used to determine DO level at reactor (JENWAY-970, England).

Field emission scanning electron microscopy (FESEM) technique for new membrane and used membrane was employed to investigate the morphology of membrane fouling due to the deposition of particle, biomass and other foulants. Also X-ray fluorescence (XRF) spectrometer was used for chemical analysis of sediment layer. To do this used membrane sheets were taken off from MBR module. Tap water was sprayed at the surface to remove any particle and attached biomass. Then membrane was cut into small pieces by sterile blades under room temperature and sent to University of Tehran laboratory for XRF analysis and instant FESEM scanning.

Silt Density Index (SDI) analyses were performed on the MBR effluents to determine the fouling potential of MBR effluent as RO influent. This test is defined by its specific procedure (ASTM D-4189). SDI is based on the time required to filter a fixed volume of water through a standard 0.45 μm pore size microfiltration membrane with a constant given pressure of 30 psi. The difference between the initial time and the time of a second measurement after normally 15 minutes (after silt-built up) represents the SDI value and calculated as below:

(1) $$\mathrm{SDI}=\frac{100\times \left(1-\frac{t_{1}}{t_{2}}\right)}{T}$$

In which:

T1= the time required to filter the first 500 mL

T2= the time required to filter the second 500 mL

T = the elapsed filtration time (normally 15 minutes) after the start of collecting the first 500 mL

## Results and discussion

### Permeate water quality of MBR

**Figure 2:**
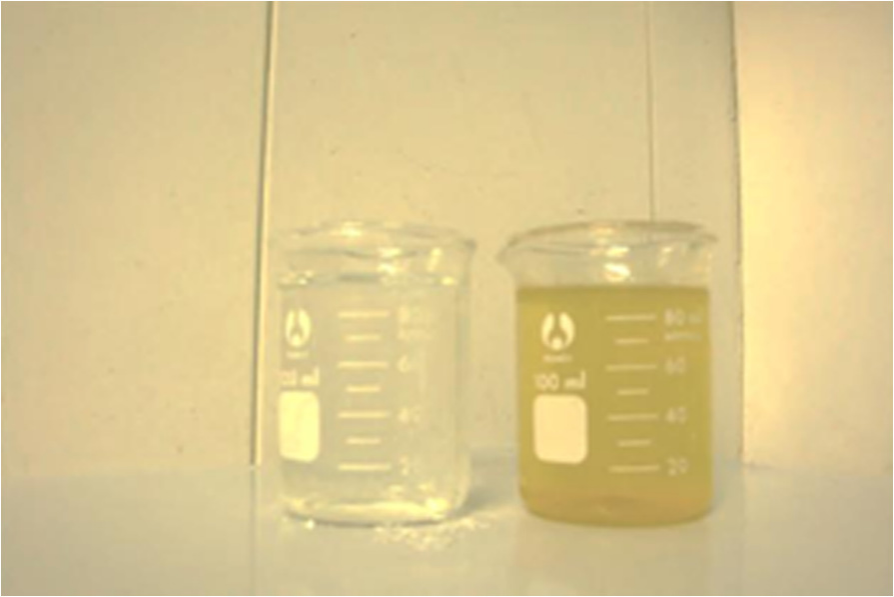
MBR mixed liquor and permeate water.

Before start the experiments, at startup phase MBR module was operated for more than 5 weeks and the stable phase was obtained. At times, values of the MLSS concentration were measured and MLSS concentration was increased up to a value of around 2000 mg/L and after that sludge removal was initiated to maintain MLSS concentration constant in the reactor. During 30 days of operation of the reactor, MBR performance base on influent and effluent quality and removal percentage data of SS, COD, TN and TC showed that system has produced permeate water with excellent quality, as was shown in Figure 2.

**Figure 3:**
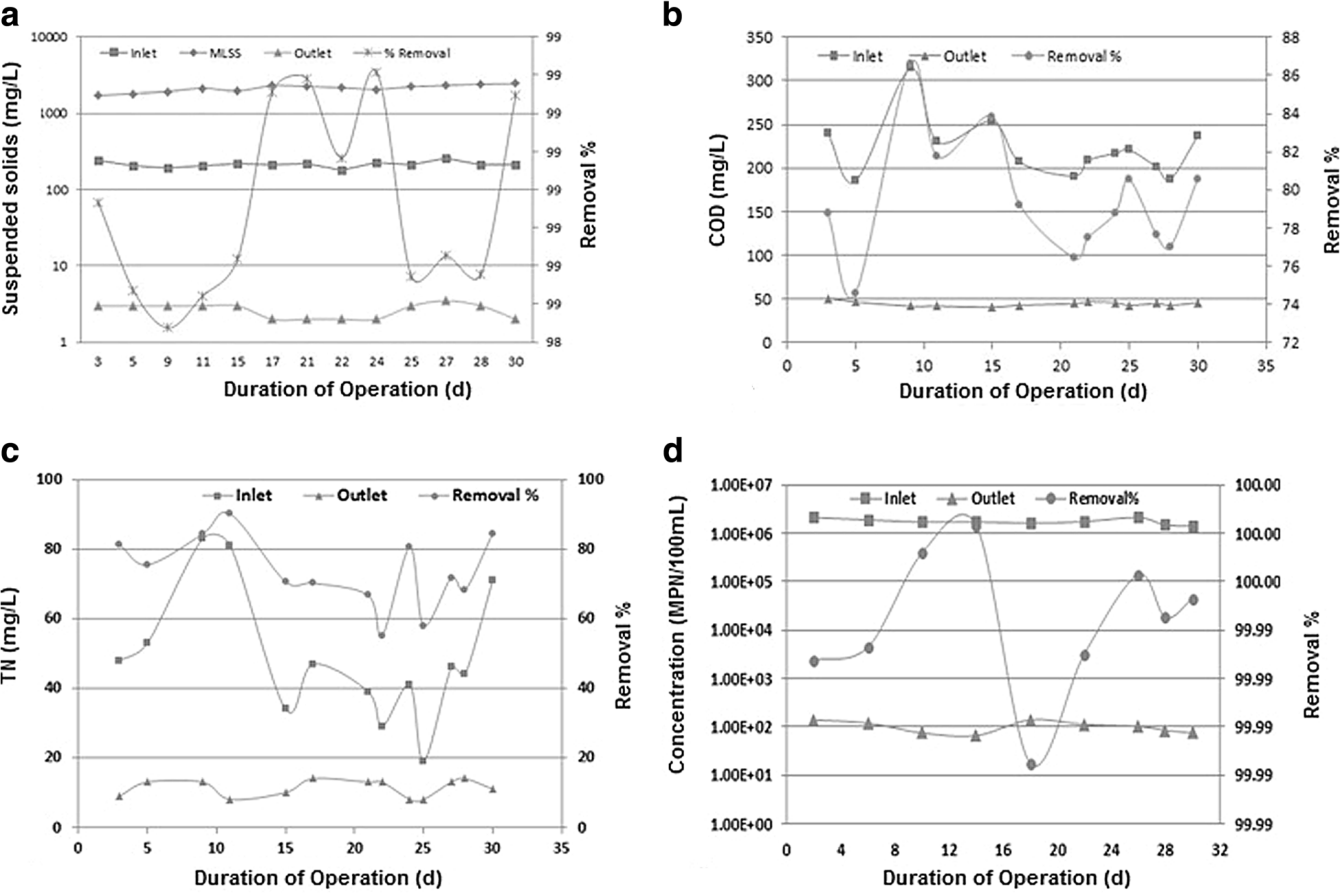
Concentration of SS, COD, TN and TC in inlet and the outlet versus the time of operation and their removal percentage.

Figure 3(a) shows the concentration of SS for inlet and outlet of MBR system and biosolids concentration (MLSS) versus the days of operation. Inlet SS concentration ranged from 179 to 243 mg/L. During this study, it was detected that MLSS of pilot was in the range of 1700–2450 mg/L. Because of the extend order of magnitudes of the concentration values, the concentration measurements are plotted on a logarithmic scale.

As in Figure 3(a) was shown excellent solids separation achieved by the UF membrane. Removal of SS reached greater than 98% resulting in the MBR permeate with SS levels below 3 mg/L.

Figure 3(b) shows the removal efficiencies of COD for influent and effluent of MBR reactor. COD was selected as indicator of organic pollution.

It can be seen that the inlet COD varied from 187 mg/L to 314 mg/L with the average COD concentration of the influent 222 mg/L whereas COD concentration in Effluent varied between 41 mg/L and 51 mg/L and average elimination rate was higher than 75%. It means MBR system has produced good removal of organic constituents and it was capable of achieving a high removal of COD and therefor organic load could be decreased effectively. Some studies reported more than 90% of COD removal which is higher than results of this study [22,23]. Lower COD removal in this study may be related to less organic material concentration in the bioreactor. Most of biodegradable organic matters are used by microorganisms during conventional treatment process. Thus, MBR feed stream (WWTP effluent) has lower amount of biodegradable matter.

TN as one of the parameters representing the content of nutrient matters is widely used in the field of wastewater treatment. Figure 3(c) depicts the TN concentrations in inlet and outlet of the MBR as a function of operation time.

As illustrated in Figure 3(c), during the experimental period, the MBR was able to reduce TN from 51±32 mg/L in raw wastewater to 11±3 mg/L on average. This means that 74% of the nitrogen was removed. The most likely reason for removal of TN should be the fact that nitrogen was removed mainly through biodegradation by microorganisms and then separation with membrane. Similar results were reported in some previous studies [24-26].

The results of total coliform analyses done on the feed wastewater and MBR permeate are shown in Figure 3(d). As results show total coliform values in the MBR effluent samples ranged from 75 to 140 MPN/100 mL, giving an overall log removal of >4 log for the total coliforms. As Figure 3(d) shows there is a trend of decreased permeate coliform with increasing time of operation. This would be expected because as the membranes become clogged the pore size is decreased which results in removal of microorganisms and other particles which could normally pass through the membrane. These data show high performance of MBRs for microorganism reduction.

### TMP trends of the MBR and membrane fouling

Membrane fouling behavior could be reflected by the developing rate of transmembrane pressure at the constant flux. As mentioned earlier this constant flux by using a prestaltic pump was set at membrane. The TMP of membrane in this study is the average driving pressure required filtering wastewater through the upper and lower membrane at the given flow rate. In the other hand the TMP is described as below:

TMP = (P_suction_ - P_permeate_) (2)

Where:

P_suction_ = Pressure measured on the suction side of the membrane

P_permeate_ = Pressure measured on the permeate side of the membrane

**Figure 4:**
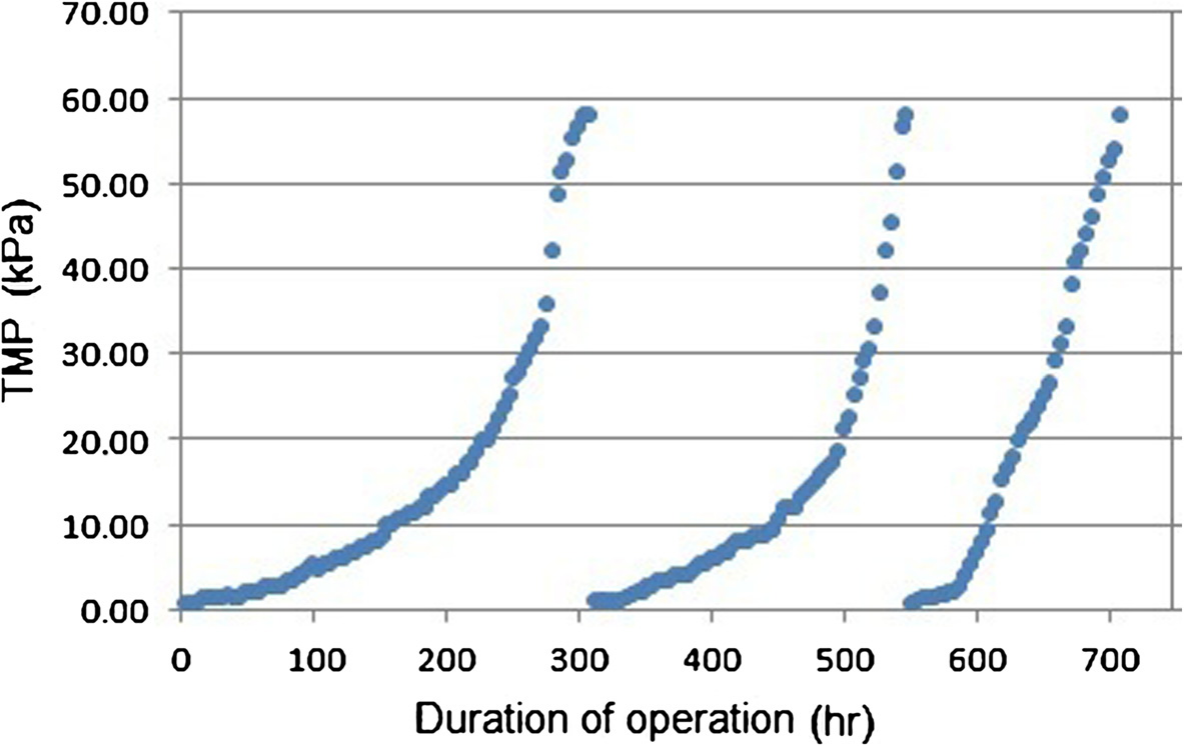
TMP changes with time.

In the experiments, the UF membrane flux was set at the constant value of 83 L/m^2^ /h and the change of TMP with time in the MBR was monitored. Figure 4 displayed the trends of flux and TMP versus the date.

It is obvious that TMP increased and went up slowly in exponential manner due to the fouling of the UF membrane. TMP reached 58 kPa on the 13th day of operation which was the fastest fouled MBR. In this stage particle, colloidal, biological and organic matters rapidly accumulated onto the membrane, and formed a cake which was probably compressible, leading to a rapid increase in the TMP. Some of these foulants are easily removed through physical wash by water, thus called reversible fouling. There is another fouling that is not readily removable from the membrane surface and requires use of chemical cleaning. As was mentioned before, for remove fouling in this study membrane was soaked in a 250 mg/L NaOCl solution and afterwards with 4000 mg/L citric acid solution for at least 4 hours. Then membrane was cleaned with tap water. However, it still remains a bit clogging of the membrane pores that are not washed away and caused pore blocking. During operation of MBR and several cleaning of membrane, pore blocking increases. Thus, as was shown in Figure 4, the time interval between the membrane washing is reduced during operation and cleaning of membrane repeats in a shorter duration.

**Figure 5:**
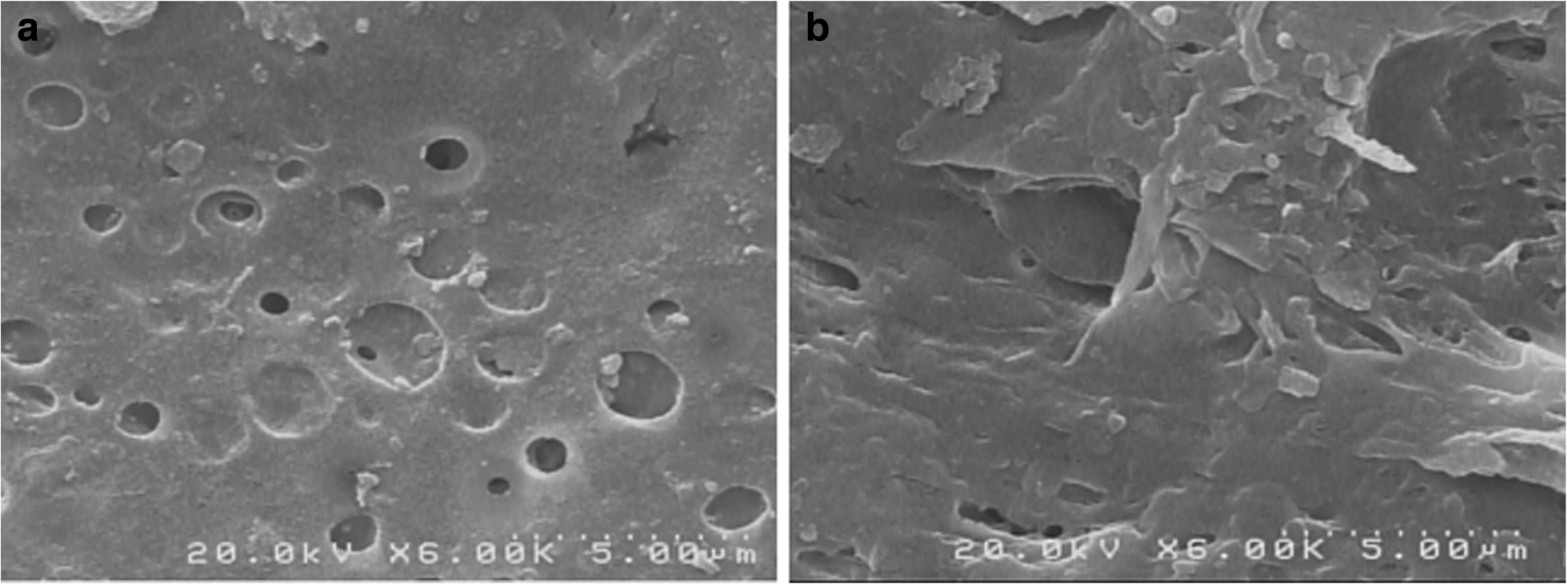
Field emission scanning electron microscopy image of membrane surface: (a) before operation; (b) after operation.

After operation, a stable bio-solids layer was formed inside membrane pores. FESEM observations of the used membrane in the reactor and a clean one were conducted at the end of the investigation to give a visual perspective of the surfaces, as shown in Figure 5.

From Figure 5, it could be seen that in comparison with the flat surface of the clean membrane, an irregular and rough gel layer was extensively distributed on the surface of the membrane in the reactor. The gel layer was found to be mainly consisted of abiotic matter which appeared to be dense and nonporous.

Results of XRF analysis of major components, expressed as percentages of the corresponding oxides, are presented in Table 4. In all samples average of components are: Na_2_O (0.379 %), MgO (0.312 %), Al_2_O_3_ (0.218%), SiO_2_ (0.838%), P_2_O_5_(0.848%), SO_3_ (0.609%), Cl (0.249%), CaO (52.222%), Fe_2_O_3_ (0.685%), Zn (0.054%) and Sr (0.286%). Other oxides are less abundant.

**Table 4:** Results of XRF analysis for foulants

**Sample**	**L.O.I.**	**Na**_**2**_**O**	**MgO**	**Al**_**2**_**O**_**3**_	**SiO**_**2**_	**P**_**2**_**O**_**5**_	**SO**_**3**_	**Cl**	**CaO**	**Fe**_**2**_**O**_**3**_	**Zn**	**Sr**
1	42.05	0.352	0.317	0.253	0.842	0.877	0.702	0.287	53.45	0.624	0.021	0.225
2	41.599	0.393	0.325	0.221	0.821	0.856	0.603	0.231	54.15	0.431	0.076	0.294
3	46.251	0.392	0.294	0.18	0.851	0.811	0.522	0.229	49.066	1.000	0.065	0.339
Ave. (%)	43.3	0.379	0.312	0.218	0.838	0.848	0.609	0.249	52.222	0.685	0.054	0.286

As it is obvious in Table 4, organic matters and calcium oxides constitute the main components of membrane fouling and contribution of other components such as heavy metals is negligible.

### SDI

**Figure 6:**
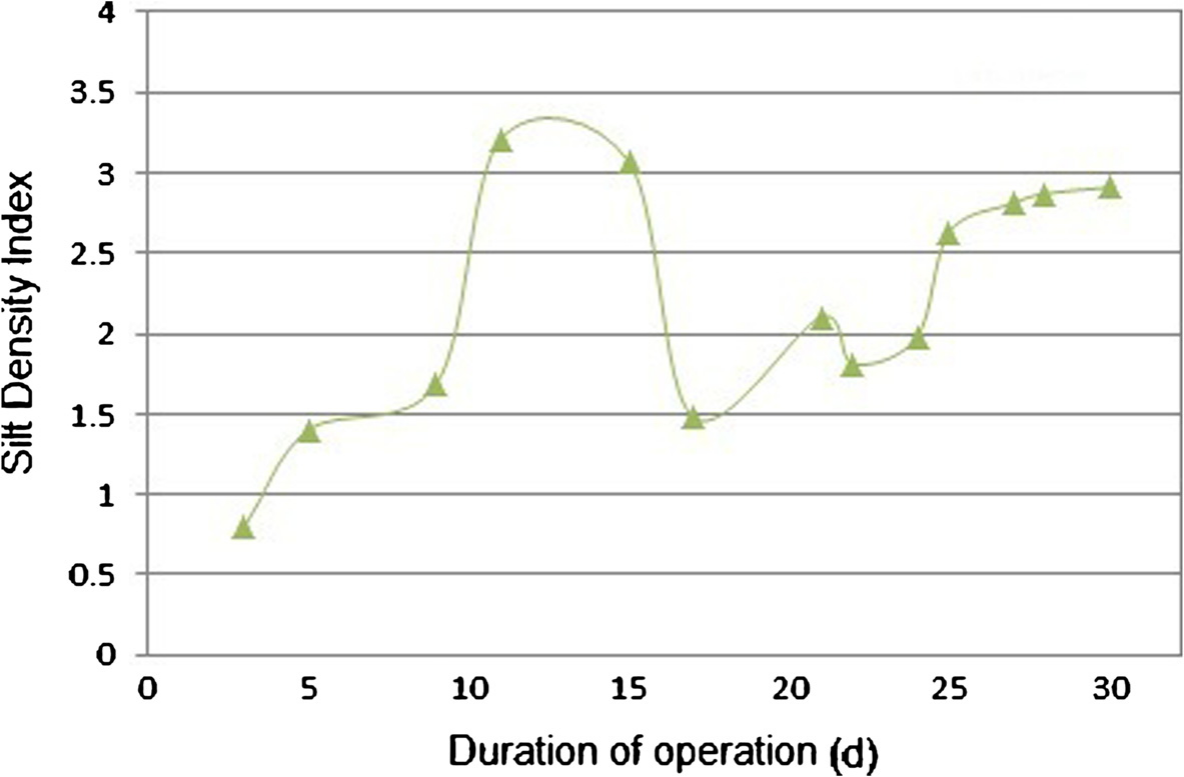
SDI trend of MBR permeate.

As mentioned before, if RO process feed directly with filtrate wastewater without any pre-treatment it will show a significant increase in process pressure. For evaluation of RO feed water quality, silt density index (SDI) measurements were taken on the MBR permeate water. Figure 6 shows the SDI trend of module permeates. The permeate SDI was below 3 for most of the time, although there was a slight increase and fluctuation during the testing periods. Measured values varied from 1.21 to 3.23, with the tendency to increase with increasing duration of operation.

In some cases the permeate SDI of the module was more than 3. High levels of organic compounds and associated biofouling, micro-air bubbles and colloidal substances are the three most likely reasons for the unexpected increase in SDI as was mentioned in previous studies [27,28]. In general SDI values of less than 3 leading to little fouling by the subsequent RO membranes. Therefore results demonstrate that MBR able to produce high quality effluent so that it would be suitable for use by an RO system.

## Conclusions

In this study the possibility and applicability of MBR to reclaim effluent of an industrial wastewater treatment plant was investigated and the MBR pilot was evaluated in terms of effluent quality. In general, it can be concluded that MBR can produce high permeate quality and is capable to be a very efficient method for RO pretreatment. Product permeate from MBR with average SDI less than 3 indicate that if MBR use as RO pretreatment, it can be anticipated that the rate of RO membrane fouling will reduce and the life of RO membrane modules will extend.

Through FESEM examinations, a gel layer was observed to be formed on the membrane surface in the MBR during the operation. Analysis of XRF results shows that organic matters and calcium oxides are the main components of membrane fouling.

Also effluent water from the MBR has a high quality according to SS, COD, TN and TC removal during operation. These results are promising and all indications show that this method is feasible for RO pretreatment and water reuse of industrial application.

## Competing interests

The authors declare that they have no competing interests.

## Authors’ contribution

MH has designed and carried out the experiments, analyzed data and wrote the manuscript. AT has conceived the strategies, developed the concept, supervised the study and finalized the manuscript. GNB and NM have guide and advice in the experiments design, suggestion of analysis and manuscript preparation. All authors read and approved the final manuscript.
